# The Role of Colon in Isolated Intestinal Transplantation: Description of 4 Cases

**DOI:** 10.1155/2024/1910430

**Published:** 2024-06-14

**Authors:** Pierpaolo Di Cocco, Giulia Bencini, Alessandro Martinino, Egor Petrochenkov, Stepan Akshelyan, Kentaro Yoshikawa, Mario Spaggiari, Jorge Almario-Alvarez, Ivo Tzvetanov, Enrico Benedetti

**Affiliations:** Department of Surgery, University of Illinois at Chicago, Chicago, IL, USA

## Abstract

Intra-abdominal desmoid tumors are a rare and complex clinical problem. These tumors are locally invasive, and surgical ablation represents the mainstay of treatment. When localized at the root of the mesentery, their resection may require extensive excision of the intestine resulting in intestinal failure and life-long total parenteral nutrition. Intestinal transplantation, either autotransplantation or allotransplantation, has been used as a viable option to treat this group of patients. Herein, we describe a series of 4 patients with unresectable intra-abdominal desmoid tumor who underwent cadaveric isolated intestinal and ascending colon transplantation.

## 1. Introduction

Familial adenomatous polyposis (FAP) is a rare genetic disorder that predisposes individuals to the development of hundreds to thousands of adenomatous polyps in the colon and rectum. This condition typically emerges during adolescence or early adulthood, with a prevalence estimated to range between 1 in 8,000 to 1 in 14,000 individuals worldwide [1].

Patients diagnosed with FAP often undergo preventive surgical procedures to lower the risk of colorectal cancer; surgical options include total colectomy with ileorectal anastomosis (IRA), total proctocolectomy with ileostomy (TPI), and restorative proctocolectomy (RPC) with or without mucosectomy and ileal-pouch anal anastomosis [2]. Moreover, approximately 10–15% of individuals with FAP develop intra-abdominal desmoid tumors [3]. These tumors, despite their benign histological characteristics, are highly invasive and frequently lead to complications such as bowel obstruction and fistula formation [4].

Surgical resection represents the treatment of choice of desmoid tumors. However, this approach can pose challenges related to their anatomical location (i.e., proximity to superior mesenteric vessels) and biological behavior (i.e., locally invasive); in selected cases, their complete resection requires extensive intestinal resection leading to intestinal failure [5]. Intestinal transplantation has been successfully employed in selected patients [6]. The surgical technique in the management of the distal graft is far from being standardized and largely depends on the recipient's anatomy.

Herein, we describe our experience on four patients with FAP and unresectable intra-abdominal desmoid tumors who underwent deceased donor-isolated intestinal and ascending colon transplantation.

## 2. Materials and Methods

We conducted a retrospective chart review and identified four patients who underwent intestinal transplantation at our institution due to intra-abdominal desmoid tumors associated with familial adenomatous polyposis (Gardner syndrome) between 2018 and 2022. All patients received isolated intestinal transplantation with en-bloc ascending colon.

Thymoglobulin, tacrolimus, and methylprednisolone were used pretransplant, and maintenance immunosuppression was achieved with tacrolimus, steroids, and basiliximab. All patients underwent regular endoscopic evaluation with biopsy posttransplant. We analyzed TPN and IVF cessation, ileostomy closure, induction and immunosuppression regimen, rejection rate and type, complications, and survival rate.

## 3. Cases Presentation

At the time of the transplant, the patient's age varied from 18 to 33 years old. The female-to-male ratio was 1 : 1. Baseline demographics are reported in Table 1.

All patients underwent prophylactic colectomy; patients 1 and 3 had total colectomy with ileorectal anastomosis, patient 2 had proctocolectomy with ileoanal anastomosis, and patient 4 had total proctocolectomy with terminal ileostomy. In two cases, patients 1 and 2, the desmoid tumor recurred at the time of the transplant, and in patients 3 and 4, the original tumor resection was performed at the time of the transplant. In all cases, the tumor engulfed the mesenteric vessels. Patient 1 underwent total enterectomy, partial gastrectomy, and right nephrectomy at the time of transplant due to desmoid tumor invasion of the antrum of the stomach and the right kidney. In this case, the intestinal continuity was restored with a side-to-side anastomosis between ascending colon of the graft and rectum. In the remaining cases, an end distal colostomy was performed.

All patients received induction immunosuppression with Thymoglobulin (antithymocyte globulin (rabbit), Genzyme Corporation) and maintenance immunosuppression with tacrolimus (Envarsus XR, Veloxis Pharmaceuticals, Inc), basiliximab (Simulact, Novartis Pharmaceuticals Corporation), and corticosteroids.

Patient 2 due to the presence of positive B crossmatch (flow and standard) required 6 sessions of plasmapheresis in addition to our standard immunosuppressive protocol. Two episodes of mild acute cellular rejection were observed in patients 1 and 2 on postoperative days 14 and 30, respectively, both successfully treated with methylprednisolone (500 mg/day for 3 consecutive days) and infliximab (5 mg/kg-single dose). Patient 1 experienced bleeding on postoperative day 3 which required exploratory laparotomy. Patient 4 presented 8 months after the transplant with parastomal hernia which required repair with mesh. No graft loss was experienced. The median follow-up period was 27 months, and the mean follow-up was 32 months, with a range of 14 to 59 months. All the recipients were alive and nutritionally independent at the time of the record review. No radiological signs of recurrence were documented.

## 4. Discussion

Intestinal transplantation has been successfully employed in the treatment of FAP patients with unresectable intra-abdominal desmoid tumors [6–8]; however, in the literature, there is no description of the technique or tactic for restoration of the distal part of the intestinal graft. This specific patient population lacks a remnant colon, which plays a crucial role in reabsorbing liquid gastrointestinal contents [9]. As a result, even with a fully functioning external sphincter, these patients often experience varying degrees of diarrhea, significantly impacting their quality of life.

Our strategy to include the ascending colon into the intestinal graft, in the selected patient population, is based on physiologic considerations of fluid reabsorption in the colon and preservation of the ileocecal valve. The ascending and transverse colon represent areas in which the ileal effluent is mixed and fermented; fluid and electrolytes are absorbed, specifically water is reabsorbed via an active sodium transport mechanism.

Ileostomies tend to produce more liquid stools with a higher risk of dehydration and renal insufficiency compared to colostomies [10]. Furthermore, the alkaline nature of stool produced by ileostomies can make the skin around the stoma more prone to irritation and inflammation [11]. Additionally, due to the relative fragility of the ileum compared to the colon, ileostomies carry a heightened risk of strictures.

Our strategy to include the ascending colon into the intestinal graft, irrespective of the fact that the bowel continuity will be reestablished, is based on the hypothesis that the presence of the colon and preservation of the ileocecal valve could yield to more solid stools, thereby reducing the risk of dehydration and local irritation. Due to these considerations, including the constrained space within the recipient's abdominal cavity, transplantation typically involves only the ascending portion of the colon [12].

The use of colon in intestinal transplantation is controversial, with some authors describing an increased risk of morbidity and mortality of the recipients, while others reporting no unfavorable impact on overall and graft survival [12, 13].

Patients with FAP and previous preventive surgical procedures to minimize the risk of colorectal cancer have two options based on their anatomy: restoration of gastrointestinal continuity with colorectal/coloanal anastomosis (patients with previous IRA and RPC/ileal-pouch anal anastomosis, respectively) or terminal colostomy (patients with previous TPI). In our series, only 1 of 4 patients (patient 1) underwent restoration of the gastrointestinal continuity with a colorectal anastomosis; in this case, a temporary loop ileostomy was performed for graft surveillance [11]. The remaining 3 patients underwent terminal colostomy; patient 2 with previous RPC/ileal-pouch anal anastomosis due to technical difficulties secondary to dense adhesions involving the anus; patient 3 with previous IRA due to patient preference; patient 4 with previous TPI.

Another important consideration is preserving the ileocecal valve into the graft, which is of paramount importance for the digestive system's proper functioning. It ensures the unidirectional flow of digested food, facilitates nutrient absorption, regulates bowel movements, and contributes to the maintenance of a gut microbiota.

To our knowledge, this is the largest series to date describing the use of ascending colon and intestinal transplant in the management of unresectable desmoid tumors in patients with FAP.

Given the uncommon nature of the disease, its wide range of anatomical sites, and the intricacy of treatment strategies, there is not a universally accepted method for conducting active surveillance or if it should be conducted at all. The latest UK guidelines recommend informing patients about the potential for postsurgery desmoid growth based on genetic factors and familial background. In such scenarios, a radiological screening is advised 12 months postoperation [14]. A recent study's initial findings indicate that typical tumor expansion might play a role in gauging disease severity. However, more research is required to validate this theory and determine a specific risk threshold or range [15].

In conclusion, the development of desmoid tumors in patients with FAP, involving the root of the mesentery, presenting local invasion, and requiring intestinal transplantation, remains an uncommon condition. There is little consensus in the literature on the management of the distal portion of the intestinal tract; the type of reconstruction depends on patient characteristics (i.e., anatomy and patient preference) and the expertise of the Institution and individual surgeon. In our experience, the inclusion of the ascending colon in an isolated small bowel graft appears to be safe; the satisfactory results in terms of continence and quality of life certainly highlight the potential for its implementation in this much selected patient population.

## 5. Limitations

The main limitation of this study is the small number of participants, consisting of only five individuals who underwent intestinal transplantation, and the limited duration of observation, which spanned 59 months. This small sample and short follow-up period may restrict the extent to which the results can be applied to a wider group of transplant recipients. Additionally, it may not fully capture long-term outcomes and potential complications that could arise after the first year following transplantation. To gain a more comprehensive understanding of the long-term safety and effectiveness of the new maintenance immunosuppression approach, a study with a larger participant pool and a longer timeframe is needed. Nonetheless, the current study offers important initial findings that merit further exploration to verify their relevance and advantages in transplant medicine.

## Figures and Tables

**Table 1:** Baseline demographics.

Baseline demographics and clinical characteristics	*N* = 4 patients
Age, years, median (range)	29 (18–33)
Male, *n* (%)	2 (50)
Race *n* (%)	
Caucasian	3 (75)
Hispanic	1 (25)
Height, cm median (range)	160 (152–173)
Weight, kg median (range)	53.7 (50.3–64)
Cause of intestinal failure, *n* (%)	
FAP—desmoid tumor	4 (100)
Crossmatch, *n* (%)	
Negative	3 (75)
Positive B (flow and standard)	1 (25)
HLA (A-B-DR) mismatch, median (range)	5 (4–5)
Peak panel reactive antibody, median (range)	
Class I	43 (0–88)
Class II	0 (0–71)
Current panel reactive antibody, median (range)	
Class I	0 (0–88)
Class II	0 (0–71)

## Data Availability

Data are available upon request. Please contact the corresponding author.

## Abbreviations

FAP: Familial adenomatous polyposis
IRA: Total colectomy with ileorectal anastomosis
TPI: Total proctocolectomy with ileostomy (TPI)
RPC: Restorative proctocolectomy
GI: Gastrointestinal
IVF: Intravenous fluids
MMF: Mycophenolate mofetil
LOS: Length of stay
TPN: Total parenteral nutrition.
